# Pharmacogenetic distinction of the Croatian population from the European average

**DOI:** 10.3325/cmj.2022.63.117

**Published:** 2022-04

**Authors:** Željka Celinšćak, Matea Zajc-Petranović, Maja Šetinc, Anita Stojanović Marković, Marijana Peričić Salihović, Hrvojka Marija Zeljko, Branka Janićijević, Nina Smolej Narančić, Tatjana Škarić-Jurić

**Affiliations:** 1 Institute for Anthropological Research, Zagreb, Croatia; 2 Faculty of Medicine, Josip Juraj Strossmayer University of Osijek, Osijek, Croatia; 3 Warrington and Halton Hospitals, NHS Foundation Trust, Warrington, United Kingdom

## Abstract

**Aim:**

To compare the Croatian and European population in terms of allele frequencies of clinically relevant polymorphisms in drug absorption, distribution, metabolism, and excretion (ADME) genes.

**Methods:**

In 429 Croatian participants, we genotyped 27 loci in 20 ADME genes. The obtained frequencies were merged with the published frequencies for the Croatian population by sample size weighting. The study sample obtained in this way was compared with the average data for the European population from the gnomAD database.

**Results:**

Variant allele frequencies in the Croatian population were higher in three and lower in two polymorphisms (Benjamini-Hochberg-corrected *P* values: 0.0027 for CYP2B6*4 rs2279343, CYP2C9*2 rs1799853, and *VKORC1* rs9923231; 0.0297 for *GSTP1* rs1695; 0.0455 for *CYP2A6* rs1801272) compared with the European population. The most marked difference was observed for CYP2B6*4 (9.3% in Europe vs 24.3% in Croatia). The most clinically relevant findings were higher variant allele frequencies in two polymorphisms related to lower warfarin requirements: VKORC1*2 (34.9% in Europe vs 40.1% in Croatia) and CYP2C9*2 (12.3% in Europe vs 14.7% in Croatia). This indicates that three-quarters of Croatian people have at least one variant allele at these loci. Variants in genes *GSTP1* and *CYP2A6* were significantly less frequently observed in Croatia.

**Conclusions:**

Croatian population has a higher bleeding and over-anticoagulation risk, which is why we recommend the prescription of lower doses of anticoagulation drugs such as warfarin and acenocoumarol. Lower phenytoin, and higher bupropion and efavirenz doses are also recommended in the Croatian population.

Drug efficacy and toxicity highly vary among individuals. This variation is a consequence of age, weight, comorbidities, diet and other environmental exposures, drug-drug interactions, and genetics. Genes responsible for absorption, distribution, metabolism, and excretion (ADME) of drugs are frequently investigated as the frequencies of their single nucleotide polymorphisms (SNP) considerably differ within and between populations (1). Drug response variability is affected by both pharmacokinetics and pharmacodynamics. Genomic knowledge can help predict individual drug response and facilitate the selection of appropriate drugs, which leads to better therapeutic response and reduces drug side effects and toxicity.

Adverse drug reactions (ADRs) cause substantial morbidity and mortality and put a strain on health care services (2). Croatia has the highest adverse drug reactions rate per one million inhabitants in the region (3). ADRs, along with efficacy and dosage, determine the clinical effect of a drug.

To our knowledge, this is the first study to compare the genetic variations in as many as 27 loci in 20 ADME genes in the Croatian population with the European data. We combined all the already published data with the data from 429 newly genotyped DNA samples and compared these data with the data in the European gnomAD database. Additionally, this study summarized the implications of the Croatian population pharmacogenetic characteristics on the pharmacotherapy practice.

## Patients and methods

### Study populations

*Croatian population*. The Croatian study sample consisted of 429 DNA samples genotyped in 2019 for 27 polymorphisms in ADME genes and the published frequencies of the same polymorphisms in the Croatian population (up to the end of 2019). Biological samples were obtained from groups of adult participants at age extremes (the age ranges being selected for a human longevity study): 327 unrelated people of both sexes aged 85 years and older (old cohort) and 102 unrelated young people of both sexes aged 20-35 years (young cohort). The allele and genotype frequencies were compared between the two groups (Supplementary Table 1 (Supplementary Table 1)and Supplementary Table 2(Supplementary Table 2)). Since no significant difference was found, the groups were merged. The participants provided informed consent, and all research methods were performed in accordance with the approved guidelines. The study was approved by the Scientific Board and Ethics Committee of the Institute for Anthropological Research.

*European population*. European population average allele frequency data were obtained from the gnomAD database (https://gnomad.broadinstitute.org/). We used data on persons who did not participate as cases in case-control studies of common diseases and who did not belong to the Finnish population. The Finnish population is a genetic isolate exhibiting unique genetic patterns caused by several founder effects and population bottlenecks (4,5), and is highly overrepresented in the European gnomAD database.

### Selection of markers and genotyping

Thirty functionally relevant ADME polymorphisms were initially selected based on examining the *PharmaADME.org* webpage and on searching all reports on ADME genes frequencies in the Pubmed database. We took into account the effect of pharmacogenetics on drug selection and dosage for the medications commonly prescribed in older age. Most of the selected polymorphisms (20 out of 30) are classified as 1A, 1B, or 2A clinical annotation levels of evidence according to the Pharmacogenomics Knowledge Base – PharmGKB, a pharmacogenomics knowledge resource that encompasses clinical information including clinical guidelines and drug labels, potentially clinically actionable gene-drug associations, and genotype-phenotype relationships (6). An additional selection criterion was remarkable population differentiation at the world-wide scale. The final list included 30 SNPs in 22 genes.

The genomic DNA was isolated from peripheral blood using the salting-out method (7). Thirty selected ADME polymorphisms were genotyped in a commercial facility with the Kompetitive Allele Specific PCR (KASP) method (8), and 27 of them were genotyped successfully.

Pharmacotherapy recommendations for the Croatian population were based on the Pharmacogenomics Knowledge Base (6).

### Data analysis

Allele and genotype frequencies were calculated with the direct counting method. Testing for Hardy-Weinberg equilibrium (HWE) was performed with Arlequin 3.5.2.2 (9). Allele frequencies for the Croatian population were calculated by weighting frequencies according to sample sizes for each SNP (pondering). If published data regarding SNP frequencies in the Croatian population were not available, only results from newly genotyped data were used. Differences between samples were tested in a pairwise fashion with the 2 × 3 χ^2^ test or Fisher´s exact test with IBM SPSS Statistics for Windows, version 21.0 (10). The results were corrected for multiple testing with the Benjamini-Hochberg procedure (11).

## Results

**Figure 1:**
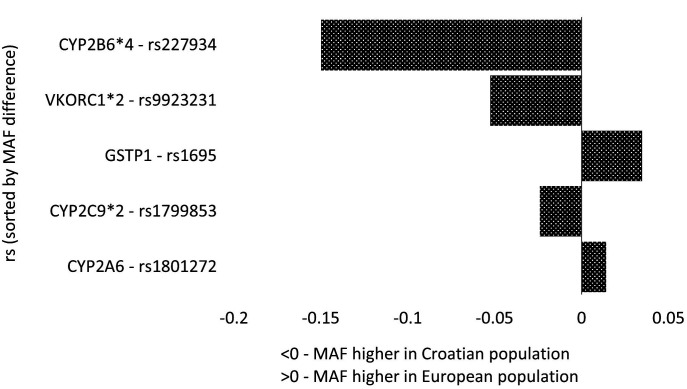
Significant variant allele frequency differences between the Croatian and average European population. To the left of zero are the variant allele frequencies that are higher in the Croatian population, and to the right of zero are variant allele frequencies that are higher in the European population. MAF – minor allele frequency.

The allele frequencies of 27 polymorphisms in 20 ADME genes in the Croatian population and in gnomAD European data are presented in Table 1. Variant allele frequencies refer to the variant alleles as indicated in the gnomAD database (12). In the Croatian population, three loci were monomorphic (rs4986893, rs5030865 and rs4148323) and 24 were polymorphic. All polymorphic loci, except rs3758581, were in the Hardy-Weinberg equilibrium. Compared with the European population, the Croatian population showed significantly higher variant allele frequencies at six investigated loci (rs2279343, rs9923231, rs1045642, rs1799853, rs28371725, and rs1057910) and significantly lower variant allele frequencies at two loci (rs1695 and rs1801272). However, after Benjamini-Hochberg correction, significant differences remained in only five loci: three showing higher frequencies in the Croatian population (CYP2B6*4, VKORC1*2, and CYP2C9*2) and two showing higher frequencies in the European population (*GSTP1* and *CYP2A6*) (Figure 1). The largest variant allele frequency difference (15%) was found for CYP2B6*4 (rs2279343), while the most clinically relevant finding was a higher variant allele frequency in the Croatian population in two polymorphisms – VKORC1*2 rs9923231 and CYP2C9*2 rs1799853 – both relevant for anticoagulant therapy.

**Table 1:** Allele frequencies and sample sizes for the 27 absorption, distribution, metabolism, and excretion (ADME) gene loci in Croatian and gnomAD European populations

Gene	Rs	Single nucleotide variant (gnomAD); chromosome - position - wide allele - variant alelle	Variant allele (gnomAD)	gnomAD (v2.1.1 controls)		Croatian population			p (Chi^2^ test)	p (BH)*
variant allele frequency	total allele number	variant allele frequency	total allele number	references		
*ABCB1*	1045642	7-87138645-A-G	G	0.4608	48272	0.4878	2348	(36-39), this study	0.0097	0.0524
*ABCB1*	1128503	7-87179601-A-G	G	0.5657	48228	0.5788	1438	(36,40), this study	0.3178	0.6129
CYP2B6*6	3745274	19-41512841-G-T	T	0.2367	48248	0.2260	838	This study	0.4858	0.6903
*ABCG2*	2231142	4-89052323-G-T	T	0.1029	48288	0.0914	1734	(40-42), this study	0.1160	0.3480
CYP1A1*2C	1048943	15-75012985-T-C	C	0.0338	48292	0.0430	844	This study	0.1781	0.4809
*CYP2A6*	1801272	19-41354533-A-T	T	0.0261	48152	0.0120	816	This study	0.0101	0.0455
CYP2B6*4	2279343	19-41515263-A-G	G	0.0932	42920	0.2430	820	This study	<0.0001	0.0027
*CYP2B6*	8192709	19-41497274-C-T	T	0.0591	48278	0.0520	826	This study	0.4559	0.7241
CYP2C19*3	4986893	10-96540410-G-A	A	0.0003	48262	0.0000	722	(43), this study	1.0000	1.0000
CYP2C19*1	3758581	10-96602623-G-A	A	0.0646	48276	0.0710	820	This study	0.4738	0.7107
CYP2C19*17	12248560	10-96521657-C-T	T	0.2503	5454	0.2392	3070	(15,40,43), this study	0.2602	0.5855
CYP2C8*3	10509681	10-96798749-T-C	C	0.1085	48290	0.1150	824	This study	0.5344	0.7214
CYP2C9*2	1799853	10-96702047-C-T	T	0.1228	48278	0.1467	4066	(15,20,40,42-44), this study	<0.0001	0.0027
CYP2C9*3	1057910	10-96741053-A-C	C	0.0676	48284	0.0764	4050	(15,20,42-44), this study	0.0379	0.1279
*CYP2D6 (* * *41* *)*	28371725	22-42523805-C-T	T	0.0887	48130	0.1056	1332	(15,43), this study	0.0302	0.1165
*CYP2D6 (* * *8* ; * *14* *)*	5030865	22-42525035-C-T	T	0.0000	45808	0.0000	838	This study	1.0000	1.0000
*CYP3A4*	2242480	7-99361466-C-T	T	0.0958	48220	0.0960	834	This study	1.0000	1.0000
*DPYD*	1801265	1-98348885-G-A	A	0.7728	48220	0.7600	828	This study	0.3799	0.6838
*GSTP1*	1695	11-67352689-A-G	G	0.3325	47974	0.2977	1586	(29,30), this study	0.0044	0.0297
NAT2*13A	1041983	8-18257795-C-T	T	0.3104	48170	0.3053	898	(43), this study	0.7709	0.9050
NAT2*6B	1799930	8-18258103-G-A	A	0.2837	48232	0.2850	824	This study	0.9379	1.0551
*SLCO1B1*	4149056	12-21331549-T-C	C	0.1606	48252	0.1735	1230	(41,43), this study	0.2385	0.5854
*SLCO1B3*	4149117	12-21011480-T-G	G	0.8461	48218	0.8510	832	This study	0.7345	0.9014
TPMT*2	1800462	6-18143955-C-G	G	0.0024	48270	0.0026	2210	(43,45), this study	0.6587	0.8469
*UGT1A1*	4148323	2-234669144-G-A	A	0.0025	48288	0.0000	828	This study	0.2759	0.5730
*UGT2B15*	1902023	4-69536084-A-C	C	0.4773	48250	0.4910	816	This study	0.4370	0.7374
*VKORC1*	9923231	16-31107689-C-T	T	0.3488	5508	0.4014	1752	(19,43), this study	<0.0001	0.0027

*Benjamini-Hochberg correction: p* = p ×27 / rank order.

**Figure 2:**
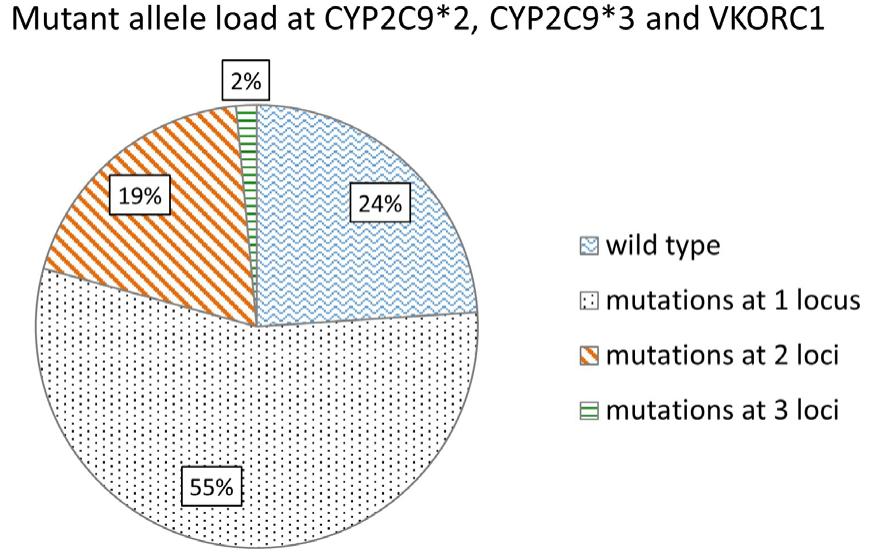
Proportion of the variant alleles in the Croatian population at CYP2C9*2, CYP2C9*3, and *VKORC1* gene loci responsible for the anticoagulant drugs metabolism.

Medication usage recommendations for the loci that significantly differed from the gnomAD database are summarized in Table 2. Variant alleles of the *VKORC1* and *CYP2C9* genes indicate the need for reduced doses of drugs such as warfarin, acenocoumarol, and phenytoin in the Croatian population compared with Europe. Overall, 76% of the Croatian population carried at least one variant allele at the three loci in CYP2C9*2, CYP2C9*3, and VKORC1*2 genes responsible for anticoagulant drugs metabolism. Specifically, 55% of participants had one variant allele, 19% had two variant alleles, while 2% had three variant alleles at different polymorphic loci (Figure 2). Additionally, allele in CYP2B6*4 indicates ultra-rapid metabolism of bupropion and efavirenz. Decreased GSTP enzyme activity in the Croatian population affects the toxicity of drugs such as cyclophosphamide and epirubicin, platinum compounds and cisplatin, but also leads to better treatment outcome when fluorouracil and oxaliplatin are used. Furthermore, due to the detected lower frequency of the variant in *CYP2A6* gene, it may be assumed that non-carriers of the polymorphism in the Croatian population metabolize nicotine better than other populations in Europe.

**Table 2:** Pharmacotherapy implications for the Croatian population based on the differences in absorption, distribution, metabolism, and excretion (ADME) gene frequencies between Croatian and gnomAD European populations

Population	Drug	Gene	SNP	Variant allele*	Phenotype	Dosing (level of evidence)	Adverse reactions (level of evidence)
CRO	warfarin	*VKORC1*	rs9923231	T ↑	Express less VKORC1 enzyme	Decreased dose of the drug is required (1A)	Increased risk of over-anticoagulation (2A)
	acenocoumarol					Decreased dose of the drug is required (2A, 1B, 2A)	Increased risk of hemorrhage (2A)
CRO	warfarin	*CYP2C9*	rs1799853	T ↑	Poor metabolizer	Decreased dose of the drug is required (1A)	Increased risk of over-anticoagulation (2A) Increased risk of bleeding (2A)
	acenocoumarol					Decreased dose of the drug is required (2A, 1B, 2A)	Increased likelihood of overcoagulation
	phenytoin						Increased toxicity and increased adverse reactions (1A)
CRO	bupropion	CYP2B6*4	rs2279343	G ↑	Ultra rapid metabolizer		Patients with the genotype AG or GG may have increased metabolism of bupropion and increased concentrations of hydroxybupropion, a metabolite of bupropion, and may require an increased dose of the drug. (2A)
	efavirenz						Patients with the AG and GG genotype and HIV may have increased clearance and decreased plasma concentration of efavirenz. (2A)
EUR	cyclophosphamide and epirubicin	*GSTP1*	rs1695	G ↓	Decreased enzyme activity		1) Decreased drug response 2) Increased severity of toxicity (2A)
	platinum compounds						Decreased, but not absent, risk of toxicity (2A)
	fluorouracil and oxaliplatin						Better treatment outcome (increased response, increased overall survival time, and reduced risk of death) (2A)
	cisplatin						Increased risk of ototoxicity (2B)
EUR	nicotine	*CYP2A6*	rs1801272	T ↓	Poor metabolizer		Decreased metabolism of nicotine (2A)

*variant allele more frequent (↑) or less frequent (↓) in Croatia (CRO) compared with gnomAD avarage (EUR).

## Discussion

In this study, the Croatian population exhibited differences from the European average in allele frequencies in several polymorphic loci. These findings indicate a need for different medication dosages or selection of alternative medications.

### CYP2C9 and VKORC1 variants

CYP2C9 is responsible for the oxidation of xenobiotic and endogenous compounds. A common *CYP2C9* non-synonymous variant (CYP2C9*2 rs1799853) leads to poor metabolism phenotypes and ADR pathogenesis. Patients with this variant frequently require lower doses of drugs with a narrow therapeutic index, such as S-warfarin, phenytoin, glipizide, and tolbutamide (13). CYP2C9 is responsible for approximately 90% of phenytoin metabolism, with the CYP2C9*2 allele decelerating the metabolism of this drug. Patients with epilepsy carrying at least one CYP2C9*2 allele may have decreased phenytoin metabolism, increased plasma phenytoin concentration, and increased ADR occurrence compared with wild-genotype patients (6). Furthermore, polymorphisms in *CYP2C9* considerably affect the pharmacokinetics and drug response variability of celecoxib, a medication used to treat osteoarthritis, rheumatoid arthritis, acute pain, and familial adenomatous polyposis. Since CYP2C9 is the main factor in the metabolic mechanism of celecoxib, CYP2C9 poor metabolizers, as well as normal metabolizers in combination with drugs that inhibit CYP2C9, have increased exposure to celecoxib (14).

Ganoci et al (15), who studied five cytochrome 450 enzyme genes (*CYP2C9*, *CYP2C19*, *CYP2D6*, *CYP3A4,* and *CYP3A5*) in 1080 participants from all parts of Croatia, found the prevalence of CYP2C9*2 allele to be 14.49%. In 23.52% of respondents, the *2 allele was part of the *1/*2 diplotype (intermediate metabolizers), while in 1.76% and 1.94% it was part of the poor metabolizing diplotypes *2/*2 and *2/*3, respectively. The polymorphisms frequencies were found to be similar to those in other European populations presented in the 1000 Genomes database and other published data on European populations (15).

Warfarin is an anticoagulant drug that reduces the activity of vitamin K-dependent clotting factors, consequently preventing blood clot formation and breaking down already formed blood clots. It is prescribed to avoid thromboembolic diseases in deep vein thrombosis, prevent embolic stroke in atrial fibrillation, and treat patients with prosthetic heart valves. The S-isomer, which is responsible for the drug's anticoagulant activity, is primarily metabolized by CYP2C9 (80%-85%) (16). Warfarin use in the Croatian population increased from 2011 to 2015 by 63% (17). *VKORC1* gene expression modifications result in fewer functional copies of the mature VKORC1 protein (18). Variant allele carriers require a lower initial dose of warfarin than do wild-type allele carriers, with additive effect. As a result, heterozygotes respond to an intermediate warfarin dose, while homozygotes require the lowest warfarin dose and are at the greatest risk of adverse events (18).

A population study by Mandić et al (19) assessed the prevalence of the rs9923231 and rs9934438 polymorphisms in the *VKORC1* gene in 420 unrelated healthy individuals from Croatia (aged 21-79 years). The prevalence of variant alleles (40.7%) did not significantly differ from that in the European white population. The authors concluded that 47% of the Croatian population carried one variant of the *VKORC1* haplotype, ie, that this portion of the population has up to 36% lower warfarin dose requirements. Persons homozygous for this variant allele (17% of the population) have up to 60% lower dose requirements. They are likely to be more prone to excessive anticoagulation and adverse events (19).

The Croatian population exhibits higher frequencies of risk variants in genes responsible for warfarin response, which may lead to the need for lower warfarin doses. In our study, further analysis of newly genotyped samples showed that 76% of the participants carried at least one variant allele related to lower warfarin requirement (CYP2C9*2, CYP2C9*3, or VKORC1*2). Specifically, 55% of participants had one variant allele, 19% had two variant alleles, while 2% had three variant alleles at different polymorphic loci. This finding indicates the possibility of a high incidence of ADRs to warfarin therapy, and points to the need for modifying therapy, especially in persons with genotypes associated with increased sensitivity (VKORC1*2/*2) and/or *CYP2C9* poor metabolism. Thus, only a quarter of the Croatian population could be treated with the standard warfarin dosage. The introduction of warfarin therapy based on a genotype-guided algorithm for patients with atrial fibrillation and ischemic stroke in Croatia reduced the required stabilization period and improved anticoagulant effectiveness (20,21).

### CYP2B6 variants

The major biotransformation enzyme CYP2B6, member of the cytochrome P450 family of relevant pharmacogenes, is responsible for the metabolism of 4% of the top 200 prescription drugs (22), including anesthetics, anticancer drugs, antidepressants, antiretrovirals, and antismoking agents (23,24). *CYP2B6* gene is one of the most polymorphic ADME genes in humans. This study found that its variant CYP2B6*4, defined by allele G of the locus rs2279343, was more frequent in the Croatian population (24.3%) than in the gnomAD European data (9.3%). CYP2B6*4 is classified as 2A clinical annotation level for bupropion and efavirenz. Patients carrying an increased function star allele (*4 and *22) in combination with an allele of normal function (*1, *2, and *5) or decreased function (*6) may have increased metabolism of bupropion and increased concentrations of hydroxybupropion, a metabolite of bupropion, in comparison with non-carriers. HIV-positive heterozygotes for the CYP2B6*4 allele manifested decreased clearance and increased plasma concentration of efavirenz when compared with patients with the wild-type genotype.

### GSTP1 variants

Glutathione S-transferases (GSTs) are enzymes that use glutathione conjugation to detoxify a variety of drugs and possible carcinogens (25). *GSTP1* variants have been linked to toxic effects and unwanted outcomes of anti-cancer drugs, especially platinum agents. A non-synonymous polymorphism *GSTP1* 313A>G is associated with decreased enzyme activity, resistance to anticancer drugs, and toxicity (26-28). A lower frequency of the variant allele in the Croatian population implies a decreased drug response and an increased toxicity of cyclophosphamide and epirubicin treatments; patients with *GSTP1* variants who are treated with platinum compounds have a lower risk of toxicity and death, as well as experience a better treatment outcome, but those treated with cisplatin have a higher risk of ototoxicity.

The rs1695 SNP of the *GSTP1* gene has been studied in the general population of Croatia to investigate its association with Alzheimer disease (29), chronic obstructive pulmonary disease (30), and multiple sclerosis (31). However, it was not studied as a pharmacogene, and its prevalence was not compared with that in non-Croatian populations. Although these three studies had varying sizes of the control group, since the studies were performed by the same authors we cannot exclude that they involved the same control population. We compared our findings with the control group in the Alzheimer disease study as the control groups in the other two studies were more than three times smaller (29). The control group in the mentioned study consisted of 231 healthy volunteers aged 18-75 years, blood donors of both sexes (161 men and 70 women), residents of Zagreb or the surrounding area. The prevalence of the variant allele in that study was 28.6%, which is similar to our 29.8%.

### CYP2A6 variants

CYP2A6 is an enzyme responsible for the metabolism of many xenobiotic compounds, whereas about 3% of drugs metabolized by CYP-450 enzymes involve CYP2A6. Variations in highly polymorphic *CYP2A6* gene alter the enzymatic activity (32). *CYP2A6* polymorphisms affect nicotine metabolism and influence the effectiveness of nicotine replacement-based smoking cessation therapies. Slow metabolizers (as defined by the CYP2A6*2, *4, *9 and *12 star alleles) are less likely to be smokers, consume fewer cigarettes per day, take smaller puff sizes, have weaker dependency, are more able to quit smoking, and benefit more from regular and extended nicotine patch replacement therapy than normal metabolizers. In contrast, alleles conferring increased enzyme activity, due to increased nicotine metabolism, are linked to increased cigarette intake and inhalation depth. The Croatian population had two times less prevalent poor-metabolizer allele CYP2A6*2, which is responsible for decreased metabolism of nicotine when combined with another poor metabolizer allele (33,34). Therefore, the Croatian population is expected to have an increased cigarette consumption and higher levels of dependence. According to a 2015 survey by the Croatian Institute for Public Health, 31.1% of the Croatian population are smokers (35).

### Strengths and limitations of the study

To our knowledge, this is the first study that presents the genotyped data on 27 polymorphisms of 20 ADME genes for the Croatian population combined with the findings of previous studies. We also compared the prevalence of the studied SNP variants in the Croatian population with the European average population and tested whether the recommended doses of medications met the needs of the local population. In addition, this study included the oldest old cohort (85+ years), whose allele frequencies in pharmacotherapeutically important polymorphisms were compared with those observed in general population of Croatia. The importance of this newly genotyped sample lies in the fact that drug safety and efficacy are particularly important in the elderly population, which is – in addition to physiological specificities related to the aging process – especially prone to ADRs as it is more often exposed to polypragmasy.

The limitation of this research is the significantly smaller number of participants in the young cohort, which necessitates that the obtained lack of significant difference between the groups is considered with caution.

In conclusion, this study showed that the Croatian population had a higher frequency of variant alleles in the two genes responsible for the metabolism of warfarin and acenocoumarol. This finding suggests a higher risk of bleeding and over-anticoagulation in the Croatian population, which warrants the use of lower doses of these medications. The results also indicate the more frequent need for lower doses of phenytoin, but higher doses of bupropion and efavirenz.
