# Supplementary material for: Pharmacogenetic distinction of the Croatian population from the European average
Source: Croat Med J. 2022 Apr;63(2):117–25. doi: 10.3325/cmj.2022.63.117 (PMC9086818; doi:10.3325/cmj.2022.63.117)
Supplement: Supplementary Table 1 [file CroatMedJ_63_s001.pdf]

Supplementary table S1: The variant allele frequencies and sample sizes for the selected 27 ADME genes' loci in two extreme adult age groups of the Croatian population (newly genotyped samples)

Gene (or star allele)	rs	Allele	Old cohort		Young cohort		Total		p
			N	frequency (%)	N	frequency (%)	N	frequency (%)	
ABCB1	1045642	A	339	54.5	95	56.5	434	54.9	0.663
		G	283	45.5	73	43.5	356	45.1	
ABCB1	1128503	G	363	57.4	119	60.1	482	58.1	0.564
		A	269	42.6	79	39.9	348	41.9	
ABCC2 (CYP2B6)	3745274	G	494	76.7	155	79.9	649	77.4	0.247
		T	150	23.3	39	20.1	189	22.6	
ABCG2	2231142	G	569	91.5	177	91.2	746	91.4	0.884
		T	53	8.5	17	8.8	70	8.6	
CYP1A1*2C	1048943	T	616	96.0	192	95.0	808	95.7	0.554
		C	26	4.0	10	5.0	36	4.3	
CYP2A6	1801272	A	611	98.5	195	99.5	806	98.8	0.466
		T	9	1.5	1	0.5	10	1.2	
CYP2B6*4	2279343	A	471	75.2	150	77.3	621	75.7	0.632
		G	155	24.8	44	22.7	199	24.3	
CYP2B6	8192709	C	594	94.9	189	94.5	783	94.8	0.855
		T	32	5.1	11	5.5	43	5.2	
CYP2C19*3	4986893	G	632	100.0	188	100.0	820	100.0	/
		A	0	0.0	0	0.0	38	0.0	
CYP2C19*1	3758581	G	579	92.5	183	94.3	762	92.9	0.427
		A	47	7.5	11	5.7	58	7.1	
CYP2C19*17	12248560	C	472	74.9	146	73.7	618	74.6	0.779
		T	158	25.1	52	26.3	210	25.4	
CYP2C8*3	10509681	T	552	87.6	177	91.2	729	88.5	0.199
		C	78	12.4	17	8.8	95	11.5	
CYP2C9*2	1799853	C	564	87.0	178	89.0	742	87.5	0.541
		T	84	13.0	22	11.0	106	12.5	

CYP2C9*3	1057910	A	597	93.6	184	94.8	781	93.9	0.610
		C	41	6.4	10	5.2	51	6.1	
CYP2D6 (*41)	28371725	C	562	89.5	176	89.8	738	89.6	1.000
		T	66	10.5	20	10.2	86	10.4	
CYP2D6 (*8; *14)	5030865	C	636	100.0	202	100.0	838	100.0	/
		T	0	0.0	0	0.0	20	0.0	
CYP3A4	2242480	C	565	89.7	189	92.6	754	90.4	0.273
		T	65	10.3	15	7.4	80	9.6	
DPYD	1801265	A	471	75.0	158	79.0	629	76.0	0.296
		G	157	25.0	42	21.0	199	24.0	
GSTP1	1695	A	440	68.8	134	69.8	574	69.0	0.859
		G	200	31.2	58	30.2	258	31.0	
NAT2*13A	1041983	C	429	68.5	131	72.0	560	69.3	0.412
		T	197	31.5	51	28.0	248	30.7	
NAT2*6B	1799930	G	452	71.1	137	72.9	589	71.5	0.647
		A	184	28.9	51	27.1	235	28.5	
SLCO1B1	4149056	T	533	83.3	161	80.5	694	82.6	0.392
		C	107	16.7	39	19.5	146	17.4	
SLCO1B3	4149117	G	538	84.6	170	86.7	708	85.1	0.493
		T	98	15.4	26	13.3	124	14.9	
TPMT*2	1800462	C	625	99.8	199	99.5	824	99.8	0.426
		G	1	0.2	1	0.5	2	0.2	
UGT1A1	4148323	G	640	100.0	188	100.0	828	100.0	/
		A	0	0.0	0	0.0	0	0.0	
UGT2B15	1902023	A	317	50.5	98	52.1	415	50.9	0.740
		C	311	49.5	90	47.9	401	49.1	
VKORC1	9923231	C	369	58.8	126	64.9	495	60.2	0.131
		T	259	41.2	68	35.1	327	39.8	

(shaded) variant alleles
