# Supplementary material for: Pharmacogenetic distinction of the Croatian population from the European average
Source: Croat Med J. 2022 Apr;63(2):117–25. doi: 10.3325/cmj.2022.63.117 (PMC9086818; doi:10.3325/cmj.2022.63.117)
Supplement: Supplementary Table 2 [file CroatMedJ_63_s002.pdf]

Supplementary table S2: Genotype frequencies and sample sizes for the selected 27 ADME genes' loci in two extreme adult age groups of the Croatian population (newly genotyped samples).

Gene	rs	Genotype	Old cohort		Young cohort		Total		p
			N	frequency (%)	N	frequency (%)	N	frequency (%)	
ABCB1	1045642	AA	84	27.0	25	29.8	109	27.6	0.873
		GA	171	55.0	45	53.6	216	54.7	
		GG	56	18.0	14	16.7	70	17.7	
ABCB1	1128503	GG	98	31.0	35	35.4	133	32.0	0.722
		AG	167	52.8	49	49.5	216	52.0	
		AA	51	16.1	15	15.2	66	15.9	
ABCC2 (CYP2B6)	3745274	GG	190	59.0	62	63.9	252	60.1	0.651
		GT	114	35.4	31	32.0	145	34.6	
		TT	18	5.6	4	4.1	22	5.3	
ABCG2	2231142	GG	258	83.0	80	82.5	338	82.8	0.879
		GT	53	17.0	17	17.5	70	17.2	
		TT	0	0.0	0	0.0	0	0.0	
CYP1A1*2C	1048943	TT	297	92.5	92	91.1	389	92.2	0.867
		TC	22	6.9	8	7.9	30	7.1	
		CC	2	0.6	1	1.0	3	0.7	
CYP2A6	1801272	AA	301	97.1	97	99.0	398	97.5	0.463
		TA	9	2.9	1	1.0	10	2.5	
		TT	0	0.0	0	0.0	0	0.0	
CYP2B6*4	2279343	AA	177	56.5	58	59.8	235	57.3	0.839
		GA	117	37.4	34	35.1	151	36.8	
		GG	19	6.1	5	5.2	24	5.9	
CYP2B6	8192709	CC	281	89.8	89	89.0	370	89.6	0.851
		CT	32	10.2	11	11.0	43	10.4	
		TT	0	0.0	0	0.0	0	0.0	
CYP2C19*3	4986893	GG	316	100.0	94	100.0	410	100.0	/

CYP2C19*1	3758581	GG	276	88.2	87	89.7	363	88.5	0.510
		GA	27	8.6	9	9.3	36	8.8	
		AA	10	3.2	1	1.0	11	2.7	
CYP2C19*17	12248560	CC	177	56.2	52	52.5	229	55.3	0.646
		CT	118	37.5	42	42.4	160	38.6	
		TT	20	6.3	5	5.1	25	6.0	
CYP2C8*3	10509681	TT	245	77.8	83	85.6	328	79.6	0.168
		TC	62	19.7	11	11.3	73	17.7	
		CC	8	2.5	3	3.1	11	2.7	
CYP2C9*2	1799853	CC	248	76.5	81	81.0	329	77.6	0.538
		TC	68	21.0	16	16.0	84	19.8	
		TT	8	2.5	3	3.0	11	2.6	
CYP2C9*3	1057910	AA	280	87.8	87	89.7	367	88.2	0.688
		CA	37	11.6	10	10.3	47	11.3	
		CC	2	0.6	0	0.0	2	0.5	
CYP2D6 (*41)	28371725	CC	254	80.9	80	81.6	334	81.1	0.978
		CT	54	17.2	16	16.3	70	17.0	
		TT	6	1.9	2	2.0	8	1.9	
CYP2D6 (*8; *14)	5030865	CC	318	100.0	101	100.0	419	100.0	/
CYP3A4	2242480	CC	255	81.0	88	86.3	343	82.3	0.469
		CT	55	17.5	13	12.7	68	16.3	
		TT	5	1.6	1	1.0	6	1.4	
DPYD	1801265	AA	181	57.6	65	65.0	246	59.4	0.414
		GA	109	34.7	28	28.0	137	33.1	
		GG	24	7.6	7	7.0	31	7.5	
GSTP1	1695	AA	148	46.2	44	45.8	192	46.2	0.703
		GA	144	45.0	46	47.9	190	45.7	
		GG	28	8.8	6	6.2	34	8.2	

NAT2*13A	1041983	CC	146	46.6	46	50.5	192	47.5	0.623
		CT	137	43.8	39	42.9	176	43.6	
		TT	30	9.6	6	6.6	36	8.9	
NAT2*6B	1799930	GG	159	50.0	48	51.1	207	50.2	0.705
		AG	134	42.1	41	43.6	175	42.5	
		AA	25	7.9	5	5.3	30	7.3	
SLCO1B1	4149056	TT	224	70.0	66	66.0	290	69.0	0.660
		TC	85	26.6	29	29.0	114	27.1	
		CC	11	3.4	5	5.0	16	3.8	
SLCO1B3	4149117	GG	232	73.0	73	74.5	305	73.3	0.389
		GT	74	23.3	24	24.5	98	23.6	
		TT	12	3.8	1	1.0	13	3.1	
TPMT*2	1800462	CC	312	99.7	99	99.0	411	99.5	0.426
		GC	1	0.3	1	1.0	2	0.5	
		GG	0	0.0	0	0.0	0	0.0	
UGT1A1	4148323	GG	320	100.0	94	100.0	414	100.0	/
UGT2B15	1902023	AA	79	25.2	26	27.7	105	25.7	0.888
		AC	159	50.6	46	48.9	205	50.2	
		CC	76	24.2	22	23.4	98	24.0	
VKORC1	9923231	CC	108	34.4	41	42.3	149	36.3	0.302
		CT	153	48.7	44	45.4	197	47.9	
		TT	53	16.9	12	12.4	65	15.8	
